# Climate Solutions Double as Health Interventions

**DOI:** 10.3390/ijerph182413339

**Published:** 2021-12-18

**Authors:** Nicholas A. Mailloux, Colleen P. Henegan, Dorothy Lsoto, Kristen P. Patterson, Paul C. West, Jonathan A. Foley, Jonathan A. Patz

**Affiliations:** 1 Center for Sustainability and the Global Environment, Nelson Institute for Environmental Studies, University of Wisconsin-Madison, Madison, WI 53726, USA; namailloux@wisc.edu (N.A.M.); henegan@wisc.edu (C.P.H.); dlsoto@wisc.edu (D.L.); 2 Project Drawdown, San Francisco, CA 94118, USA; kristen.patterson@drawdown.org (K.P.P.); paul.west@drawdown.org (P.C.W.); jfoley@drawdown.org (J.A.F.); 3 Department of Applied Economics, University of Minnesota, St. Paul, MN 55108, USA; 4 Global Health Institute, University of Wisconsin-Madison, Madison, WI 53706, USA; 5 Department of Population Health Sciences, University of Wisconsin-Madison, Madison, WI 53726, USA

**Keywords:** climate change, climate mitigation, energy, health benefits, air quality, physical activity, diet and nutrition, infectious disease, voluntary family planning, universal education

## Abstract

The climate crisis threatens to exacerbate numerous climate-sensitive health risks, including heatwave mortality, malnutrition from reduced crop yields, water- and vector-borne infectious diseases, and respiratory illness from smog, ozone, allergenic pollen, and wildfires. Recent reports from the Intergovernmental Panel on Climate Change stress the urgent need for action to mitigate climate change, underscoring the need for more scientific assessment of the benefits of climate action for health and wellbeing. Project Drawdown has analyzed more than 80 solutions to address climate change, building on existing technologies and practices, that could be scaled to collectively limit warming to between 1.5° and 2 °C above preindustrial levels. The solutions span nine major sectors and are aggregated into three groups: reducing the sources of emissions, maintaining and enhancing carbon sinks, and addressing social inequities. Here we present an overview of how climate solutions in these three areas can benefit human health through improved air quality, increased physical activity, healthier diets, reduced risk of infectious disease, and improved sexual and reproductive health, and universal education. We find that the health benefits of a low-carbon society are more substantial and more numerous than previously realized and should be central to policies addressing climate change. Much of the existing literature focuses on health effects in high-income countries, however, and more research is needed on health and equity implications of climate solutions, especially in the Global South. We conclude that adding the myriad health benefits across multiple climate change solutions can likely add impetus to move climate policies faster and further.

## 1. Introduction

The latest report of the Intergovernmental Panel on Climate Change (IPCC) concluded that it is now unequivocal that heating of the planet is caused by human activities and that the 1 °C of warming above preindustrial times currently being observed is already disrupting weather in every region of the planet [1]. Climate change poses many risks to human health, and numerous climate-sensitive health risks are scientifically established [2]. Continued warming of the planet will lead to increasingly dangerous extreme weather events (such as heat waves, floods, droughts, and wildfires), cause significant sea level rise, have dramatic effects on ecosystems and natural resources, and threaten human wellbeing worldwide [3,4].

The urgency for timely action previously emerged from the IPCC’s Special Report on Global Warming of 1.5 °C, in which climate scientists determined the emissions reductions needed to stabilize the planet’s temperature at 1.5 °C above preindustrial levels. The IPCC concluded that limiting global warming to 1.5 °C would require a 45% reduction in greenhouse gas (GHG) emissions by 2030 (relative to 2010 levels), followed by reaching net zero emissions by 2050 [3]. Efforts to reduce, or mitigate, climate change will require emissions reductions in all sectors. However, the majority of climate solutions in public policies and awareness are focused on the electricity and transportation sectors. These two sectors combined account for 39% of global greenhouse gas emissions [5]. Yet, solutions across all sectors are essential to meeting 2030 emission reduction and 2050 net-zero targets.

Founded in 2014, Project Drawdown is a nonprofit organization that seeks to help the world reach “drawdown”—the future point in time when levels of greenhouse gases in the atmosphere stop climbing and start to steadily decline. Project Drawdown has undertaken a comprehensive assessment of the effectiveness, scale, and cost of scores of climate mitigation measures [5]. Their system of solutions spans all sectors, exist today, have proven potential to reduce GHGs in the atmosphere, and are financially viable. Project Drawdown’s research shows that if existing solutions are brought to scale in the coming decades, we could halt global warming between the 2040s and 2060s.

The changes we must make to address climate change will require profound shifts in cultural, political, technological, and economic systems worldwide, while concurrently— and dramatically—scaling up economic development and access to quality health and education in low- and middle-income countries. If implemented poorly, these changes could reinforce existing health inequities between and within countries [6,7]. Solutions to the climate crisis can provide “win–win” opportunities for public health, but it is crucial that such efforts center equity and social justice. Past work has identified and reviewed key health benefits in the realms of air quality, physical activity, and diets, among others [8,9,10,11,12,13]. Climate adaptation efforts aimed at reducing vulnerability to climate change impacts represent a vast and important topic area, but they are beyond the scope of this perspective, which focuses on health benefits of climate mitigation strategies. Original research on health and climate change has increased 11-fold from 2007 to 2020 [4], and there is evidence to suggest that highlighting the health benefits of climate and clean energy policy can increase public support for such policy [14,15,16].

Building from a priority list of climate solutions created by Project Drawdown, we review information on a wide array of health benefits that can accompany climate mitigation. We aim to provide a summary of the dominant health themes that surround climate solutions, which could be of use to academics, practitioners, and others engaged in research and communication on climate and health. In particular, health professionals are becoming increasingly involved in climate change advocacy. This group ranks among the most trusted groups in society and has an essential role to play in promoting solutions to the climate crisis [17,18,19]. However, health professionals report a need for resources to help convey the breadth of health benefits that climate solutions offer [20]. The purpose of this perspective is to leverage the strengths of Project Drawdown’s comprehensive analysis of climate mitigation solutions with research on the linkages between climate, infrastructure, education, and public health. These insights provide a foundation for evidence-based policies that are effective at both mitigating and addressing climate change while also improving human wellbeing.

## 2. Climate Solutions Cut across Many Sectors

**Figure 1:**
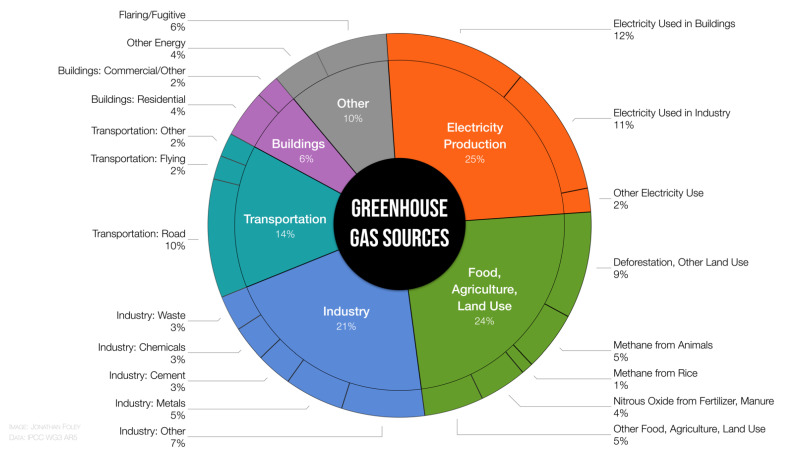
Emissions of greenhouse gases by sector and area of human activity. Emissions are weighted by their global warming potential over a 100-year period. Data are from the Working Group III Contribution to the Fifth Assessment Report of the IPCC [21].

Human activities release several planet-warming emissions including carbon dioxide, methane, nitrous oxide, fluorinated gases, black carbon, and others. The world’s greenhouse gas emissions result from numerous activities across six major sectors: electricity production; food, agriculture, and land use; industry; transportation; buildings; and other sources (Figure 1) [5]. These emissions result from fossil fuel combustion for energy use as well as from agricultural and land use processes, industrial processes, and other sources. The loss of forests and other carbon “sinks” also lessens the ability of the earth’s surface to sequester carbon dioxide from the atmosphere.

**Figure 2:**
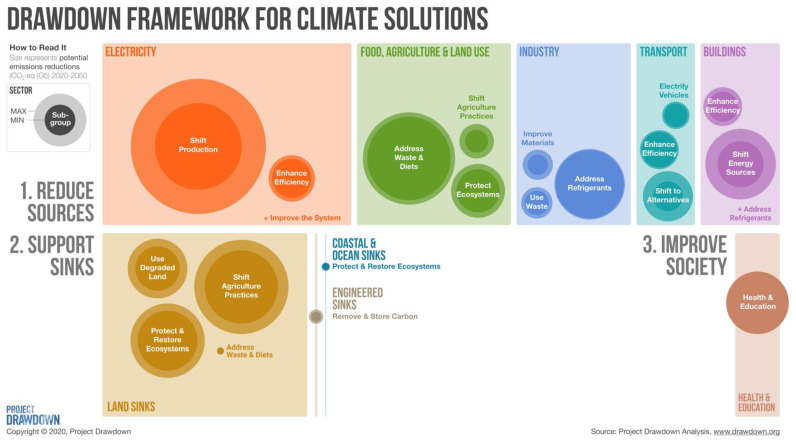
Climate solution thematic areas. Climate solutions are divided into three major categories: those that reduce sources of pollution, those that enhance sinks of carbon removal, and those that address inequities in society with cascading benefits for climate. Minimum and maximum values represent the potential emissions reduction or sequestration of each sector from 2020 to 2050 under two different implementation scenarios, which roughly align with goals of limiting global temperature rise to 2° and 1.5 °C, respectively. For more details about the underlying methodology, see The Drawdown Review [5]. Reproduced with permission from Project Drawdown.

Project Drawdown has proposed more than 80 climate solutions in three large areas spread across nine major sectors (Figure 2). The first area of solutions focuses on reducing sources of greenhouse gases, which represent the majority of potential emissions reductions. The second set of solutions aims at maintaining and enhancing carbon sinks, especially those linked to nature, on land and in the oceans. The third area of solutions centers actions that reduce inequities in society—human rights issues concerning education and health—that can have ancillary benefits for climate. Here we present an overview of how climate solutions proposed by Project Drawdown in these three areas can benefit human health through improved air quality, increased physical activity, healthier diets, reduced risk of infectious disease, and improved sexual and reproductive health, and universal education (Table 1 and Table 2).

## 3. Improved Air Quality

Ambient fine particulate matter (PM_2.5_) pollution is the leading environmental risk factor for disease globally [22]. Largely the result of fuel combustion for residential energy use, industry, and electric power generation [23], ambient PM_2.5_ pollution is responsible for more than 4 million premature deaths each year [24], though other analyses have suggested the toll is even greater. One recent study put the total at close to 9 million [25], and a recent study estimates that ambient PM_2.5_ pollution from fossil fuel combustion alone led to 8.7 million premature deaths in 2018 [26].

Shifting energy use for electricity generation, transportation, buildings, and industry away from combustible fuel sources—such as coal, natural gas, petroleum, wood, and dung—and toward cleaner alternatives could greatly reduce greenhouse gas emissions and help reduce the burden of disease from air pollution.

Replacements of coal and natural gas with wind and solar for electricity generation from 2007 and 2015 in the United States were found to have prevented 3000–12,700 premature deaths through lowered emissions of PM_2.5_, sulfur dioxide, and nitrogen oxides [27].

In countries that continue to rely heavily on coal for electricity production, switching to renewable sources could have profound health benefits. One study estimated that fossil fuel-related PM_2.5_ and ozone (O_3_) pollution lead to nearly 1.6 million excess premature deaths per year in China and 690,000 in India [26].

Vehicle emissions in the transportation sector are a significant source of PM_2.5_ and O_3_ pollution [28]. Electrifying vehicles could greatly reduce the air pollution burden from transportation by eliminating vehicle exhaust emissions. One study estimated that reducing transportation emissions in the United States by 75% by 2030 could prevent 14,000 premature deaths each year from reduced PM_2.5_ and O_3_ exposure [29]. Similarly, electrifying 27% of China’s private vehicle fleet by 2030 could prevent 17,500 premature deaths annually from improvements in air quality [30]. Several studies have demonstrated that air pollution from vehicle traffic emissions disproportionately affects people of color and low-income populations, indicating that climate solutions aimed at reducing vehicles emissions could also improve health and equity [31,32].

Non-exhaust emissions—from brake wear, tire wear, road wear, and suspension of road dust—also comprise a considerable portion of total road vehicle emissions [33]. There is evidence to suggest that replacing an internal combustion engine vehicle (ICEV) with an electric vehicle (EV) could actually increase non-exhaust emissions since EVs tend to weigh more than similar ICEVs and would release more tire wear and dust resuspension emissions per vehicle mile [34]. Tire and brake wear can also contribute to the emission of heavy metals such as iron, zinc, copper, and nickel that can lead to heavy metal toxicity [35]. Alternate transportation-related climate solutions, such as carpooling, public transit, and high-speed rail, could provide pathways for further reducing transportation emissions.

One of the most dramatic health benefits from addressing climate change comes from shifting fuel sources for indoor cooking and heating. Nearly 800 million people, three-quarters of whom live in sub-Saharan Africa, do not have access to electricity [36]. About three billion people rely on solid fuels—including charcoal, coal, crop waste, dung, and wood—for cooking and heating [37]. Household air pollution from the burning of solid fuels is the second greatest environmental risk factor for disease globally—exceeded only by ambient PM_2.5_ pollution—and leads to more than 2 million premature deaths each year [22,24].

Many studies have shown that using cleaner cookstoves can decrease household air pollution exposure and improve respiratory and cardiovascular health outcomes through fuel switching and increased ventilation [38,39]. One global analysis estimated that in countries where more than 5% of the population uses solid fuels for cooking, switching to clean cooking could prevent more than 22.5 million premature deaths between 2000 and 2100 from avoided ambient PM_2.5_ exposure [40].

However, select systematic reviews and meta-analyses show that while some cookstove intervention programs have resulted in decreased exposure to pollutants such as particulate matter and carbon monoxide, evidence is mixed regarding the extent to which such programs improve health outcomes [41,42,43]. Two reviews of the health effects of cookstove interventions proved inconclusive [41,42] and a third found that improved biomass cookstoves had no significant effect on child health outcomes, including lower acute respiratory infections and severe pneumonia, but did lead to reductions in chronic obstructive pulmonary disorder (COPD), numerous respiratory symptoms, and conjunctivitis among women in low- and middle-income countries [43]. The overall evidence is in favor of clean cooking solutions that rely on electricity or clean-burning liquefied petroleum gas (e.g., propane) to realize their climate mitigation and health benefits potential [44,45,46]. These solutions would also provide myriad additional benefits around income and work, education, and gender equality, particularly for women and girls, who are often responsible for cooking and solid fuel collection [47].

While poor air quality affects populations around the world, the highest mortality rates from ambient PM_2.5_ pollution are found in China, India, and parts of Eastern Europe while death rates from household air pollution are highest in many sub-Saharan African countries and some South and South-East Asian countries [24]. The loss of life expectancy from household air pollution is 0.7 years but is more than two years in several sub-Saharan African countries, for example [48].

In high-income countries such as the United States, clean air policies have improved air quality, yet disparities in air pollution exposure still exist, with low-income communities and communities of color often exposed to relatively worse air quality [49,50]. One review of the health equity implications of air pollution control strategies in Europe notes that since susceptible groups—such as the elderly, children, pregnant women, and groups with pre-existing health conditions—often have higher baseline mortality rates than the general population, a given reduction in pollution exposure can provide a greater benefit to these groups, thus increasing health equity [51]. Several studies highlight examples of how to assess health and equity impacts of air quality policies [52,53] and explore potential pitfalls for assessments and interventions, such as lack of data to identify key target areas and effects on different disadvantaged groups [54,55]. Most research exploring equity impacts of air pollution is based in the United States and Europe, indicating a need for more research on equity in developing nations [56].

## 4. Increased Physical Activity

Some climate solutions call for changing transportation systems and rethinking urban design to reduce dependence on fossil-fuel powered vehicles and accommodate increased adoption of active transportation modes, such as cycling and walking. These goals can be accomplished by building cycling and walking infrastructure, expanding public transit access, and revising zoning laws to allow for high-density and mixed-use development [57]. Within the Project Drawdown framework, these goals are organized into three subgroups: shifting to alternatives, enhancing efficiency, and electrifying vehicles (see Table 2).

Numerous factors related to the built environment can influence physical activity levels, including residential housing density, street connectivity, mixed land use, the quality of active transport infrastructure, and distance to public transit stops [58,59]. One study of six cities on five continents—Melbourne, Australia; Boston, United States; London, United Kingdom; Copenhagen, Denmark; São Paulo, Brazil; and Delhi, India—found that a compact cities model in which land-use density and diversity were increased, distance to public transit was decreased, and a modal shift from private vehicle use to cycling and walking occurred would result in reductions in cardiovascular disease, respiratory disease, and diabetes [60].

For those able to replace vehicles trips with walking or bicycling, these increased opportunities for physical activity can both benefit health and reduce greenhouse gas emissions. Low physical activity is among the top behavioral risk factors for disease globally [24]. One study estimated that existing healthy lifestyle behaviors, measured by physical activity prevalence, already avert about 4 million deaths each year worldwide [61]. Studies from across the globe—from New Zealand to the Netherlands—show that replacing vehicle trips with active transportation modes can improve health by reducing cardiovascular disease, diabetes, and impaired mental health [62]. A study of the 50 US states and large US cities concluded that higher rates of walking and cycling for commuting were associated with lower levels of obesity, which can put individuals at increased risk of diabetes, hypertension, cardiovascular disease, and other illnesses [63].

The promotion of active transportation modes and public transit use requires substantial alterations to the structure and function of cities. Without careful consideration of the equity implications of such changes, these interventions can reinforce patterns of residential segregation, gentrification, and displacement of low-income residents and communities of color. One review of transit-oriented development—an urban design practice intended to maximize walking, cycling, and public transit—stresses the importance of requiring affordable housing around such development and involving local communities at early stages of development to ensure that the benefits of such projects are experienced by all [64].

## 5. Improved Nutrition and Food Security

Significant changes in the food and agriculture sector can help address climate change, particularly through the reduction of food waste and a shift to more plant-based diets. These adjustments can reduce emissions from mineral fertilizer production and application, land clearing activities, and livestock cultivation, and would provide substantial health benefits by improving nutrition and food security. Food insecurity, malnutrition, and other dietary issues are among the largest contributors to the global burden of disease and result in nearly 8 million premature deaths each year [24]. The effects of food insecurity and malnutrition come in many forms, including insufficient caloric intake, micronutrient deficiency, and overnutrition and obesity in part due to overconsumption of processed foods [65].

About one-third of food produced for human consumption is lost or wasted [66]. Food loss, which occurs during production, handling, and storage, is more common in low-income countries. Food waste is more common in higher income countries and occurs at the end of the supply chain at the retail or consumer level [66]. Reducing food loss and waste throughout the supply chain would ensure that a greater portion of food products remain available for human consumption. This would also reduce the amount of land and resources needed for food production that are significant sources of greenhouse gases, including methane from cattle and rice production, nitrous oxide from fertilizer, in addition to methane from organic landfill waste.

The health and climate benefits of plant-rich diets are well-studied. A dietary shift that increases consumption of fruits and vegetables while decreasing red meat consumption could prevent 5.1 million premature deaths each year by 2050 [67]. Global adoption of vegetarian and vegan diets would prevent even more deaths annually, at 7.3 million and 8.1 million, respectively. Similarly, the EAT-Lancet Commission found that shifting to a universal healthy reference diet—rich in fruits, vegetables, whole grain, legumes and low in red meat—could help prevent about 11 million deaths per year by 2050 [68]. These dietary shifts would also reduce emissions from ruminant animals such as beef and dairy cattle, which are the primary sources of on-farm greenhouse gas emissions from food production [69]. The health benefits of reduced red meat consumption may be more pronounced in high-income countries where excess meat consumption is common and should not overshadow the fact that livestock provide an essential nutrient source for people in many low- and middle-income countries [70].

## 6. Reduced Risk of Emerging Infectious Disease

Efforts to protect ecosystems can reduce greenhouse gas emissions, sequester carbon, bolster biodiversity conservation, and reduce emerging infectious disease risk [71,72]. Moreover, the avoided destruction of forests would maintain the capacity of some of nature’s most productive carbon “sinks” to remove carbon dioxide from the atmosphere. Recognizing land tenure and forest management rights for women, the rural poor, and Indigenous peoples can lead to better forest protection as well as benefit human livelihoods and wellbeing [73,74].

The maintenance of healthy forest ecosystems can help to prevent the emergence of novel zoonotic diseases, including bat-borne viruses such as Hendra virus. Altered bat migration patterns have been linked to deforestation across Indonesia, for example, and similar ecological processes are suspected for coronaviruses [75]. At the time of this writing, SARS-CoV-2, the virus that can lead to COVID-19, had caused more than 5.2 million deaths worldwide [76]. Forest fragmentation can increase the risk of exposure to other zoonoses such as Ebola virus disease, which has killed more than 13,000 people since it was first identified in Africa in 1976 [77]. Outbreaks of Ebola between 2004 to 2014 originated from spillover cases that occurred in forests in Central and West Africa that were significantly more fragmented than average [78]. The loss of dense forests in the same region was associated with significantly more Ebola outbreaks within two years after deforestation occurred [79].

Healthy forests also reduce the risk of vector-borne disease transmission from arthropods like mosquitoes and other insects. These disease vectors can be strongly affected by loss of forest cover, either through changes in microclimate, local patterns of biological diversity, or other environmental factors. Several studies have identified the connection between deforestation and increased malaria incidence in the Amazon region [80,81]. In the Peruvian Amazon, mosquitoes capable of transmitting malaria were significantly more common in areas with little (0% to 20%) remaining forest coverage and scarce in areas with more than 60% forest coverage. In addition, sites in the Peruvian Amazon with low forest cover and high grassland or cropland cover had a biting rate 278-fold higher than sites that were mostly forested [82].

This association between malaria and deforestation can be seen around the globe. In Indonesia, the risk of a malarial outbreak increases 2% to 4.6% per 1000 hectares of lost forest cover, resulting in an additional 45,000 to 110,000 additional infected individuals within the nation each year [83]. In Uganda, the replacement of natural swamp vegetation with cultivated swamps is associated with significantly higher minimum and maximum local temperatures and elevated malaria transmission risk [84].

## 7. Improved Sexual and Reproductive Health and Universal Education

Two society-based solutions—voluntary family planning and universal, high-quality primary and secondary education—can address societal inequities, have clear health and economic benefits, and provide ancillary benefits for climate change.

While demographers and climate scientists have long noted the link between population growth and climate change at a global scale [85], there is an opportunity to more fully embrace the cascading benefits of voluntary family planning as a climate solution through rights-based programmatic and policy interventions that ensure every person can choose whether, when, with whom, and how often to have children [86]. There are close links between educational attainment, use of family planning services, and fertility [87], though some call for additional research on the causal relationship of education on sexual and reproductive health outcomes [88]. Meeting desired fertility needs globally can generate myriad secondary benefits for climate change, including slowing future population growth [65].

Rights-based family planning decisions are founded upon full, free, and informed choice, through health care services that are available, accessible, acceptable, and of the highest possible quality [89]. The benefits of contraception for maternal and child health, nutrition, economic development, achieving the UN Sustainable Development Goals, gender equality, resilience, and planetary health are well-established [66,89,90,91]. For example, meeting the contraceptive and maternal care needs of women in low- and middle-income countries could prevent nearly three quarters of maternal deaths, with similarly dramatic decreases in newborn mortality [85]. Furthermore, there is increasing evidence that family planning decreases vulnerability to environmental shocks and stressors such as flooding, drought, and food and water scarcity, and boosts resilience [86,90].

High-quality, universal education is an essential human right with measurable benefits for health, income generation, and empowerment [92,93]. Researchers have estimated that increases in educational attainment globally among reproductive-age women from 1970 to 2009 prevented 4.2 million deaths among children younger than five years [92]. In a study of the health benefits of secondary education, researchers found that each additional year of secondary education was associated with decreases in HIV prevalence among all adolescents, but especially in young women, particularly in South Asia, Latin America, and sub-Saharan Africa [92]. There is also increasing evidence of the role of education—specifically girls’ education—in building adaptive capacity to climate-related extreme events [94]. Educated women can better protect themselves and their families from environmental shocks and are better able to participate in decision-making [95]. An analysis from 125 countries shows that education—particularly female education—is the key socioeconomic factor associated with a reduction in vulnerability to natural disasters [96]. Through climate-informed education, students learn environmentally-focused low-carbon economy job skills—an avenue to addressing social inequities, gender imbalances, and climate change at once [97,98].

The convergence of promoting sexual and reproductive health and rights, improving access and quality of education, and developing climate solutions provides an opportunity to improve the lives of women and girls while simultaneously unleashing cascading benefits for combatting climate change.

## 8. Other Health Benefits of Climate Solutions

Climate mitigation measures can provide other opportunities to improve human health and wellbeing. Examples include reducing exposure to environmental extremes, improving water quality, and improving mental health.

### 8.1. Reduced Exposure to Environmental Extremes

Restoring and conserving coastal and terrestrial ecosystems, many of which serve as carbon sinks, can benefit health by reducing risk of exposure to natural hazards such as coastal and inland flooding, extreme heat, and storm surge associated with cyclonic activity [72]. Coastal wetlands such as mangroves and marshes provide protection against storm surge flooding to 29% of the world’s coastal plains, which shield an estimated 13.5 million people from flooding impacts, 80% of whom live in China, Vietnam, the Netherlands, India, and Germany [99]. Urban greening can mitigate the urban heat island effect and provides climate mitigation benefits by decreasing energy demand for building cooling [100].

### 8.2. Improved Water Quality

The use of unsafe water sources is among the top environmental risk factors for disease globally and contributes to more than 1 million premature deaths each year, primarily due to diarrheal diseases [24]. Protecting and restoring freshwater and coastal ecosystems can safeguard stores of “blue carbon,” the carbon stored in coastal and marine ecosystems, and bolster carbon sequestration. In addition, these ecosystems improve water quality and reduce water-borne disease by removing particles, pathogens, and excess nutrients through filtration [72]. For example, higher upstream forest cover along rivers has been associated with improved water quality and reduced diarrheal disease incidence downstream in rural areas in 35 countries [72]. The presence of seagrass meadows—an important carbon sink—have been found to reduce the relative abundance of disease-causing bacterial pathogens by 50% in coastal regions of Indonesia [101].

### 8.3. Improved Mental Health

Access to nature and proximity to green space have been associated with improved mental health. Protecting and restoring ecosystems, particularly in and nearby populated areas, increases carbon storage and could help to bolster these mental health benefits. Higher levels of neighborhood greenness, measured by vegetation density, can reduce self-reported levels of distress, anxiety, depression, and other health outcomes [65]. This is an active area of research, but evidence to date is clear that contact with nature increases psychological wellbeing and reduces risk factors and burdens of some types of mental illness [102].

## 9. Conclusions

There are numerous pathways by which climate change mitigation measures can promote human health and wellbeing. These climate–health solution pathways include: reducing the combustion of fossil fuels and solid fuels to improve air quality; transforming our transportation systems to promote physical activity; altering the food and agriculture system to reduce food waste and promote more plant-rich diets; protecting ecosystems, particularly forests functioning as carbon sinks, to reduce the risk of emerging infectious diseases; and finally, providing access to voluntary family planning services and universal, high-quality education worldwide—key human rights issues of our time—which improves sexual and reproductive health and provides economic and social opportunities.

We have highlighted major health benefits of climate mitigation policies. While some areas are well-explored, such as the air quality benefits from renewable energy generation, literature on connections to health are more limited in other areas. As this perspective is intended to be readily accessible, we do not conduct a systematic analysis of all available literature. Much of the existing literature focuses on health effects in high-income countries. More research is needed on health and equity impacts of climate solutions, especially to explore impacts for communities in the Global South. The scale, timing, and distribution of health benefits of climate solutions will depend on numerous factors, including rates of technology adoption and infrastructure buildout, the acceptability and affordability of proposed technologies, and the extent to which equity considerations are included.

Addressing these challenges will require sustained coordination by governments, private industry, civil society groups, philanthropic donors, and individuals. Our collective ability to realize climate and health goals can be supported or undermined by mediating factors including governance, wealth, philanthropy, technology, culture, and behavior [103]. Equity must be central to these efforts. There are promising examples of programs that advance equity while promoting health and climate goals [104]. The risk of reinforcing existing health disparities or producing new adverse unintended consequences is too high to leave to chance.

Drawdown Lift, a new program of Project Drawdown launched in early 2021, works to deepen understanding of the links between climate change solutions, health, and improving human well-being, particularly in emerging economies in sub-Saharan Africa and Asia. Drawdown Lift works to break down disciplinary walls and find solutions that can address climate change and extreme poverty while enhancing human well-being. Lift synthesizes knowledge and encourages decisionmakers and policymakers to deploy holistic solutions to global climate and human well-being challenges.

The win–win climate and health solutions summarized here offer promising areas to focus attention as we address some of the most vexing, intertwined, and urgent challenges facing humanity. Though not the focus of this perspective, these solutions can and should be coupled with adaptation efforts to reduce vulnerability to unavoidable future climate change impacts and those already manifest today. As decision-makers learn of the substantial near-term human health benefits that can be realized by reducing greenhouse gas emissions, such health framing of solutions to the global climate crisis adds impetus to move mitigation policies faster and further.

## Figures and Tables

**Table 1:** Summary of health benefits associated with general health themes.

Health Theme	Health Benefits
Improved air quality	Improved cardiovascular and respiratory health (e.g., lower incidence of heart disease, stroke, lung cancer, diabetes, chronic obstructive pulmonary disease, pneumonia)
Increased physical activity	Reduced cardiovascular disease, diabetes, and impaired mental health; lowered risk of obesity-related illness
Improved nutrition and food security	Improved cardiovascular health (e.g., lower incidence of heart disease, stroke, and diabetes)
Reduced risk of emerging infectious disease	Reduced risk of exposure to zoonotic and vector-borne disease (e.g., Hendra virus, Ebola virus, and malaria)
Reduced exposure to environmental extremes	Reduced exposure to natural hazards (e.g., coastal and inland flooding, extreme heat, storm surge from cyclone activity)
Improved water quality	Reduced risk of water-borne disease (e.g., diarrheal disease) and toxics exposure
Improved mental health	Reduced prevalence of stress, depression, and anxiety
Improved sexual and reproductive health	Gender equality; reduced maternal, newborn, child, and adolescent mortality; reduced vulnerability to environmental stressors and climate-related extreme events
Universal education	Improved health, empowerment, climate adaptation, and resilience; reduced risk of HIV infection

**Table 2:** Linkages between health themes and Project Drawdown climate solutions.

Area	Sector and Subgroup	Climate Solutions	Health Themes
**Reduce Sources**	*Electricity*		
	Shift production	Distributed solar photovoltaics; utility-scale solar photovoltaics; onshore wind turbines; offshore wind turbines; geothermal power; biomass power; nuclear power	Improved air quality
	Enhance efficiency	Smart thermostats; building automation systems; LED lighting; insulation; green and cool roofs; high-efficiency heat pumps; solar hot water; building retrofitting	Improved air quality
	*Transportation*		
	Shift to alternatives	Walkable cities; bicycle infrastructure; electric bicycles; carpooling; public transit; high-speed rail	Improved air quality; increased physical activity
	Enhance efficiency	Hybrid cars; efficient trucks; efficient aviation; efficient ocean shipping	Improved air quality
	Electrify vehicles	Electric cars; electric trains	Improved air quality
	*Buildings*		
	Enhance efficiency	See: *Electricity*	
	Shift energy sources	Biogas for cooking; improved clean cookstoves	Improved air quality
	*Food, agriculture, and land use*		
	Address diets and waste	Plant-rich diets; reduced food waste	Improved nutrition and food security
	Protect ecosystems	See: *Land sinks*	
	Shift agriculture practices	Nutrient management; farm irrigation efficiency	Improved water quality
**Support Sinks**	*Land sinks*		
	Address waste and diets	Plant-rich diets; reduced food waste	Improved nutrition and food security
	Protect and restore ecosystems	Forest protection; indigenous peoples’ land tenure; temperate forest restoration; tropical forest restoration; grassland protection; peatland protection and rewetting	Reduced risk of emerging infectious disease; reduced exposure to environmental extremes; improved water quality; improved mental health
	*Coastal and ocean sinks*		
	Protect and restore ecosystems	Coastal wetland protection; coastal wetland restoration	Reduced exposure to environmental extremes
**Improve Society**	*Health and education*		
	Health and education	Voluntary, rights-based family planning; universal, high-quality education	Improved sexual and reproductive health; universal education

Note: The sectors and climate solutions in this table represent a partial list of solutions analyzed by Project Drawdown. For a full list of solutions and more details, see The Drawdown Review [5].

## Data Availability

No new data were created or analyzed in this study. Data sharing is not applicable to this article.
